# Coloration and the Genetics of Adaptation

**DOI:** 10.1371/journal.pbio.0050250

**Published:** 2007-09-18

**Authors:** Nicholas I Mundy

## Abstract

Coat color is often used as camouflage and so has evolutionary benefit. How is coat color determined?

How do organisms adapt to new environments? The popular conception of the adaptive process generally runs like this: A mutation arises that leads to an improved phenotype under novel environmental conditions (for example, a beak modification suited to exploiting an untapped food source). Because of the enhanced reproductive success of individuals carrying the mutant allele (or gene variant), the frequency of the mutant allele increases. The mutant allele eventually becomes fixed in the population—that is, every member of the population has two copies of the allele—and the population has increased its fitness. The whole process can then repeat itself when the environment changes again.

This is the simplest possible model of adaptation, and is enshrined in well-known examples such as the evolution of melanism in the peppered moth and of insecticide resistance in mosquitoes. However, it has long been known that there are many potential complications to this tidy scenario involving a single mutant allele at a single genetic locus, and that the situation is frequently more complex in the real world. For example, more than one locus may be involved, mutant alleles may have effects on more than one aspect of the phenotype (a phenomenon known as pleiotropy), and the effects of alleles on the phenotype may be dependent on the environment (genotype × environment interactions). The timing of the occurrence of a mutation in relation to an environmental change in which the mutation is favored—that is, whether the beneficial mutation arose de novo in the new environment or was part of the standing genetic variation—also turns out to be critically important. Recent theoretical [1] and empirical work [2–4] has greatly increased our understanding of the adaptive process, but there are many fundamental questions remaining. In particular, we have little knowledge of the relative importance of different factors in actual cases of adaptation, and there are many details of the molecular basis of adaptation that are poorly understood.

If two or more loci are involved in adaptation to a new environment, then this leads to the possibility that the phenotypic effects of the two loci are not additive, that is, they are not independent of one another. For example, the effect on the phenotype of the presence of allele A rather than allele B at one locus may depend on whether allele X or allele Y are present at a second locus. Such epistatic effects may have a profound influence on the evolutionary dynamics of adaptation. One possibility is that genetic divergence among different populations that becomes fixed for alternate alleles at one or more loci may lead to different propensities for future evolution [5]. Epistasis also plays an important role in causing genetic incompatibilities that underlie reproductive isolation and speciation. However, in contrast to the growing number of examples in which the molecular genetics of single loci underlying adaptation have been investigated, we have little information on how epistasis works at the single gene level in adaptation in the wild.

Coloration is rapidly becoming established as a phenotype that is highly amenable to studying the molecular basis of adaptation. There are several reasons for this. Color variation is abundant in natural populations and can be readily quantified. The selective advantages of coloration are widely studied and include physical protection (e.g., against ultraviolet light), thermoregulation, concealment (including background color matching and camouflage), and a large variety of signaling functions both within and between species. Finally, genetic studies on model organisms such as fruit flies and mice have led to an excellent understanding of the gene networks involved in generating certain types of coloration, especially for those genes involved in regulating the production of melanin pigments.

Recent studies on organisms ranging from flowering plants [6] to fruit flies [7,8], butterflies [9], fish [10], lizards [11], birds [12,13], and mammals [14–17] have identified single loci contributing to color variation in natural populations. Perhaps the most notable conclusion from these studies is that, with some exceptions, it is surprisingly common for the same single loci or genomic regions to be responsible for similar color changes in different species. In vertebrates, many studies have found convergent association between variation in the coding sequence at the *Mc1r* locus and melanin-based coloration. *Mc1r* encodes the melanocortin-1 receptor, which has a critical function in determining the type of melanin synthesized in hair or feather melanocytes. However, these studies may present a biased picture of the overall importance of *Mc1r* in evolution since the locus is relatively easy to assay (it has a single coding exon of ~1 kb). In laboratory mice, the *Agouti* locus regulates pale ventral coloration and pale bands on dorsal hairs [18]. *Agouti* encodes *agouti* signaling protein, an inhibitor of *Mc1r*, and ventral-specific or hair-cycle specific expression of *Agouti* diverts the melanin synthesis pathway from brown or black eumelanin to yellow phaeomelanin. Many alleles of *Agouti* have been described, but unlike *Mc1r* the great majority of them are in the large 5′ regulatory regions of the *Agouti* gene, and thus far more difficult to assay. A priori, the *Agouti* gene is as good a candidate gene as *Mc1r* for involvement in evolutionary change of coloration, but until now association between *Agouti* variation and color in wild populations has not been described.

New research published in *PLoS Biology* from the lab of Dr. Hopi Hoekstra uncovers a persuasive example where color variation in natural populations is partly controlled by epistasis among two loci, and also provides the first case where *Agouti* has been implicated in natural color variation. Steiner et al. studied two subspecies of the oldfield mouse in the southeastern United States, Peromyscus polionotus subgriseus, a mainland subspecies with dark dorsal and pale ventral coloration, and P. p. leucocephalus, which inhabits white sand dunes and lacks visible coloration over much of the body, with a narrow band of darker coloration dorsally. The color of the mice matches the color of the substrates they inhabit, and previous work has shown that this provides crypsis against avian predators.

The authors use a powerful combination of methods to identify genes underlying the phenotypic variation. Using crosses between the two subspecies in captivity, they performed a quantitative trait locus (QTL) analysis with a total of 124 genetic markers. QTL analysis is an extension of standard genetic linkage mapping (originally designed for single locus Mendelian traits) to quantitative traits and can identify genomic regions contributing to heritable variation in a trait as well as the proportion of the trait variance attributable to a particular genomic region. In Steiner et al.'s analysis, the genomic regions identified were consistent across the seven color traits they measured: two regions were significantly associated with all seven traits while a third region was associated with four traits but not the remaining three.

All three QTL regions identified contained pigmentation genes (*Mc1r, Agouti*, and *Kit*), which are therefore candidates for functional involvement in the color differences. For *Mc1r* this was no surprise since the group had already shown that an amino acid substitution at *Mc1r* is associated with coloration and that there is a functional effect of the mutation [19]. Steiner et al. investigated *Agouti* further, and, interestingly, while there was no variation in the amino acid sequence, they did find significantly higher expression of *Agouti* mRNA transcript in several body regions of the paler P. p. leucocephalus than in P. p. subgriseus. This finding is consistent with the action of *Agouti* and, although more complicated scenarios cannot be ruled out, the simplest explanation is that there are one or more mutations in the large upstream regulatory regions of the *Agouti* locus that underline the QTL discovered.

The most novel part of the study is the demonstration of epistasis occurring between the phenotypic effects of alleles at *Mc1r* and *Agouti*. At both loci, parental alleles can be classed as dark (D) in P. p. subgriseus and light (L) in P. p. leucocephalus. For all seven traits, the effects of D and L alleles at *Mc1r* are dependent on the genotype at *Agouti*. The authors present the most dramatic case, which involves cheek pigmentation. For this trait, variation at *Mc1r* has no effect on a DD genetic background at *Agouti*, whereas on a LD or LL *Agouti* background, the LL *Mc1r* genotype is paler than DL or DD (see their Figure 5). This could have strong evolutionary significance, because it means that for cheek color the L *Mc1r* allele is neutral on a DD *Agouti* background, and only becomes visible to natural selection on a pale environmental substrate in an LD or DD *Agouti* genotype. There is interesting variation in the pattern of epistasis among traits—for example, for tail color, the LL *Mc1r* genotype is visible against a DD *Agouti* background, complicating attempts to reconstruct the evolutionary sequence of events of selection on *Agouti* and *Mc1r* genotypes.

This study should stimulate further research into a number of questions. Is it generally the case that variation in color traits can be attributed to a relatively small number of loci, and is coloration different from other traits in this regard? How common is epistasis among color traits, and is it possible to predict the evolutionary consequences of particular epistatic interactions? What is the relative importance of different loci in evolutionary color change, and why? For *Agouti*, we finally have a case where the locus appears to be controlling evolutionary change in coloration—an exciting finding, given its important function in color patterning.

## Abbreviations

D: dark
L: light
QTL: quantitative trait locus
